# 3D whole-heart phase sensitive inversion recovery CMR for simultaneous black-blood late gadolinium enhancement and bright-blood coronary CMR angiography

**DOI:** 10.1186/s12968-017-0405-z

**Published:** 2017-11-27

**Authors:** Giulia Ginami, Radhouene Neji, Imran Rashid, Amedeo Chiribiri, Tevfik F. Ismail, René M. Botnar, Claudia Prieto

**Affiliations:** 1 School of Biomedical Engineering and Imaging Sciences, King’s College London, St Thomas’ Hospital (Lambeth Wing), Westminster Bridge Rd, London, SE1 7EH UK; 2 MR Research Collaborations, Siemens Healthcare Limited, Sir William Siemens Square Frimley, Camberley, GU16 8QD UK; 3 Escuela de Ingeniería, Pontificia Universidad Católica de Chile, Vicuna Mackenna, 4860 Santiago, Chile

**Keywords:** Whole-heart, Black-blood, Bright-blood, Late gadolinium enhancement (LGE), Coronary MR angiography

## Abstract

**Background:**

Phase sensitive inversion recovery (PSIR) applied to late gadolinium enhancement (LGE) imaging is widely used in clinical practice. However, conventional 2D PSIR LGE sequences provide sub-optimal contrast between scar tissue and blood pool, rendering the detection of subendocardial infarcts and scar segmentation challenging. Furthermore, the acquisition of a low flip angle reference image doubles the acquisition time without providing any additional diagnostic information. The purpose of this study was to develop and test a novel 3D whole-heart PSIR-like framework, named *BOOST*, enabling simultaneous black-blood LGE assessment and bright-blood visualization of cardiac anatomy.

**Methods:**

The proposed approach alternates the acquisition of a 3D volume preceded by a T_2_-prepared Inversion Recovery (T_2_Prep-IR) module (magnitude image) with the acquisition of a T_2_-prepared 3D volume (reference image). The two volumes (T_2_Prep-IR BOOST and bright-blood T_2_Prep BOOST) are combined in a PSIR-like reconstruction to obtain a complementary 3D black-blood volume for LGE assessment (PSIR BOOST). The black-blood PSIR BOOST and the bright-blood T_2_Prep BOOST datasets were compared to conventional clinical sequences for scar detection and coronary CMR angiography (CMRA) in 18 patients with a spectrum of cardiovascular disease (CVD).

**Results:**

Datasets from 12 patients were quantitatively analysed. The black-blood PSIR BOOST dataset provided statistically improved contrast to noise ratio (CNR) between blood and scar when compared to a clinical 2D PSIR sequence (15.8 ± 3.3 and 4.1 ± 5.6, respectively). Overall agreement in LGE depiction was found between 3D black-blood PSIR BOOST and clinical 2D PSIR acquisitions, with 11/12 PSIR BOOST datasets considered diagnostic. The bright-blood T_2_Prep BOOST dataset provided high quality depiction of the proximal coronary segments, with improvement of visual score when compared to a clinical CMRA sequence. Acquisition time of BOOST (~10 min), providing information on both LGE uptake and heart anatomy, was comparable to that of a clinical single CMRA sequence.

**Conclusions:**

The feasibility of BOOST for simultaneous black-blood LGE assessment and bright-blood coronary angiography was successfully tested in patients with cardiovascular disease. The framework enables free-breathing multi-contrast whole-heart acquisitions with 100% scan efficiency and predictable scan time. Complementary information on 3D LGE and heart anatomy are obtained reducing examination time.

**Electronic supplementary material:**

The online version of this article (10.1186/s12968-017-0405-z) contains supplementary material, which is available to authorized users.

## Background

Late gadolinium enhancement (LGE) cardiovascular magnetic resonance (CMR) imaging has become the gold standard for the assessment of myocardial viability in different cardiac pathologies, including myocardial infarction [1, 2] and myocarditis [3–5]. In addition, LGE imaging provides pre-interventional assessment of arrhythmogenic substrate in patients undergoing electrophysiology procedures as well as visualization of lesions after ablation [6–8], and is gaining importance in the characterization of fibrosis in non-ischemic cardiomyopathies [9–12]. LGE imaging is typically performed 10–20 min after the administration of a gadolinium (Gd)-based contrast agent using T_1_-weighted inversion recovery (IR) sequences [1, 13–15]. The inversion time (TI) is normally set to null the signal from the healthy myocardium, thus enhancing the contrast to noise ratio (CNR) between viable and diseased myocardial tissue. IR sequences, however, are prone to reduced scar to blood and scar to remote myocardium contrast when a sub-optimal TI is selected. Phase-sensitive IR (PSIR) LGE acquisitions have been introduced to provide intrinsic robustness with respect to the TI selection [16]. Conventional PSIR sequences are based on the acquisition of an IR-prepared image (referred to as “magnitude image”), interleaved with a proton density image (referred to as “reference image”) that is acquired at a low flip-angle, which are then combined as described in [16]. Although PSIR normally achieves excellent contrast between viable myocardium and scar tissue, the contrast between blood pool and LGE uptake is often suboptimal. This leads to difficulties in delineating sub-endocardial infarcts that are adjacent to the blood pool. Furthermore, unclear borders between scar tissue and blood affect the accuracy of scar segmentation that is crucial for infarct size and transmurality measurements as well as for the planning of electrophysiology procedures [17, 18]. Black-blood PSIR LGE has been introduced [19] to improve the contrast between the blood pool and scar tissue by exploiting an inversion pulse in combination with a T_2_ preparation (T_2_Prep) module (T_2_Prep-IR) [19–21]. However, a limitation of all PSIR frameworks is that the acquisition efficiency is intrinsically sub-optimal as the low flip-angle reference image has limited diagnostic value. Furthermore, most of the LGE PSIR implementations are limited to 2D acquisitions that are performed during a breath-hold to minimize respiratory motion artefacts. Recently, free-breathing whole-heart PSIR acquisitions have been introduced [22, 23] and integrated with diaphragmatic navigator gating [24]. The use of diaphragmatic navigator gating, however, leads to reduced scan efficiency and unpredictable acquisition times that can make the selection of the correct TI challenging. In addition, residual imaging artefacts may be observed as a result of the combination of the inversion pulse with the diaphragmatic navigator [25]. In order to overcome these drawbacks, we propose the extension of a 3D whole-heart Bright-blood and black-blOOd phase SensiTive inversion recovery (BOOST) sequence [26] – that has been recently introduced for non-contrast enhanced visualization of coronary lumen and thrombus – to black-blood LGE imaging. The proposed post-contrast BOOST sequence exploits a T_2_Prep-IR module for the acquisition of the magnitude image, enabling black-blood LGE PSIR reconstruction. Furthermore, the acquisition of the reference image is designed to provide a complementary and fully co-registered bright-blood dataset for the visualization of the heart anatomy, the great vessels, and the coronary lumen. The entire framework has been integrated with image-based navigation [27] to achieve 100% scan efficiency and predictable scan time. In this study, the feasibility of BOOST for post-contrast simultaneous black-blood LGE imaging and bright-blood heart anatomy, great vessels, and coronary lumen visualization was tested in a cohort of cardiovascular patients at the end of a clinical CMR examination.

## Methods

### Framework implementation

**Fig. 1:**
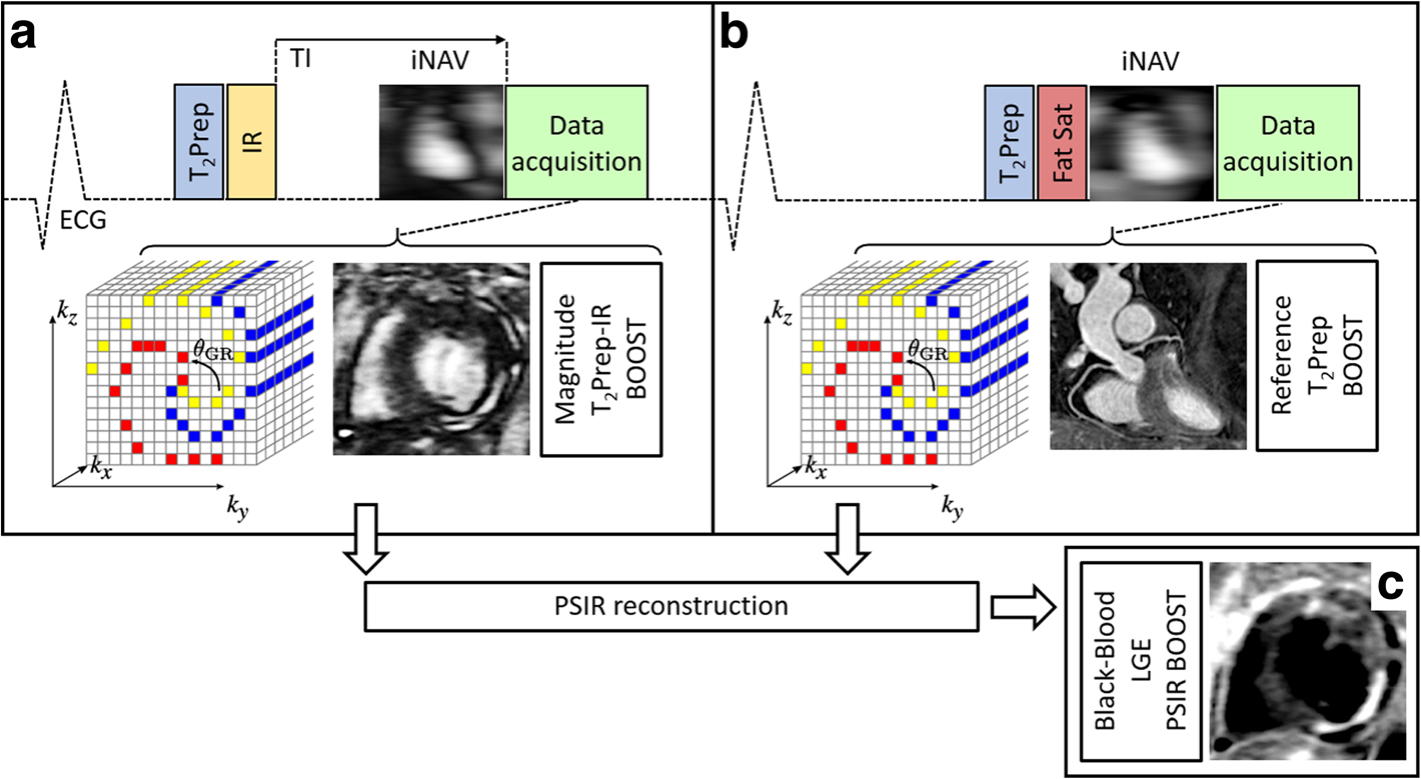
Proposed post-contrast BOOST framework for simultaneous 3D whole-heart bright-blood coronary angiography and black-blood late gadolinium enhancement (LGE) assessment. A T_2_-prepared inversion recovery (T_2_Prep-IR) module is applied at odd heartbeats (T_2_Prep-IR BOOST, magnitude image) (**a**), whereas data acquisition is T_2_ prepared and performed with a high flip angle at even heartbeats (bright-blood T_2_Prep-BOOST, reference image) (**b**). A 3D Cartesian trajectory with spiral profile order [28] is used for data acquisition; data collection is segmented over multiple heartbeats (yellow, red, blue) to minimize the effects of cardiac motion. Even heartbeat acquisitions include a SPIR pulse for fat saturation, while a STIR-like fat suppression is employed in odd heartbeats. 3D data acquisition at each heartbeat is preceded by a low-resolution 2D image-based navigator (iNAV) that is used to estimate translational respiratory motion along the superior-inferior and right-left directions. The two motion corrected datasets (T2Prep-IR BOOST and T2Prep BOOST) are combined in a PSIR-like reconstruction to generate a third, complementary, black-blood dataset (PSIR BOOST) for LGE visualization (**c**). The motion corrected bright-blood T_2_Prep BOOST dataset (reference image, **b**) provides adequate contrast for heart anatomy, great vessel, and coronary lumen visualization

An electrocardiogram -triggered free-breathing 3D whole-heart balanced steady-state free precession (bSSFP) prototype sequence was implemented as described in [26] and as illustrated in Fig. 1. The sequence alternates the acquisition of a T_2_-prepared IR volume in odd heartbeats (T_2_Prep-IR BOOST, magnitude image) and a T_2_-prepared volume in even heartbeats (bright-blood T_2_Prep BOOST, reference image). Both acquisitions are performed with a Cartesian trajectory with spiral profile order [28] and with a high flip-angle of 90 degrees. A 2D low-resolution image-based navigator (iNAV) [27] is acquired at each heartbeat to estimate translational respiratory motion along the superior-inferior (SI) and right-left (RL) directions and to enable beat-to-beat motion correction with 100% scan efficiency and predictable scan time. Prior to data acquisition, a rectangular region of interest (ROI) is selected covering the whole heart along the RL direction and covering the base and the mid part of the heart along the SI direction. RL and SI translational motion is then estimated using a template-matching algorithm [29] and motion compensation is performed by modulating the k-space data with a linear shift before image reconstruction [27]. Motion estimation is performed for the T_2_Prep-IR BOOST and T_2_Prep BOOST datasets independently. For each dataset, respiratory motion correction is performed at the end-expiratory level [30].

The motion corrected T_2_Prep-IR BOOST and T_2_Prep BOOST volumes are then rigidly co-registered and combined in a PSIR-like reconstruction as described in [16] to obtain a complimentary PSIR BOOST black-blood volume. The PSIR-like reconstruction is performed using the T_2_Prep BOOST dataset as reference image. As the T_2_Prep BOOST is designed for coronary lumen visualization, it exhibits high tissue contrast. Thus, voxel-by-voxel intensity normalization of the resulting PSIR BOOST volume by the reference image is not performed in order to preserve adequate contrast in the resulting black-blood LGE volume.

All acquisitions reported in this study were performed on a 1.5 T CMR system (Magnetom Aera, Siemens Healthineers, Erlangen, Germany) using an 18-channel chest-coil and a 32-channel spine coil. The study was approved by the National Research Ethics Service (15/NS/0030) and written informed consent was obtained from each participant according to institutional guidelines. Motion estimation and correction, image reconstruction, and PSIR-like computation were implemented using the scanner software (Syngo MR E11A, Siemens Healthineers).

### Sequence simulations

The longitudinal magnetization behavior of healthy viable myocardium, scar and blood was investigated via simulations for the proposed post-contrast BOOST sequence. This was compared against the corresponding simulations for previously published LGE PSIR and bright-blood coronary angiography post-contrast sequences. Sequence simulations were performed in Matlab R2016a (The MathWorks, Inc., Natick, Massachusetts, USA).

**Fig. 2:**
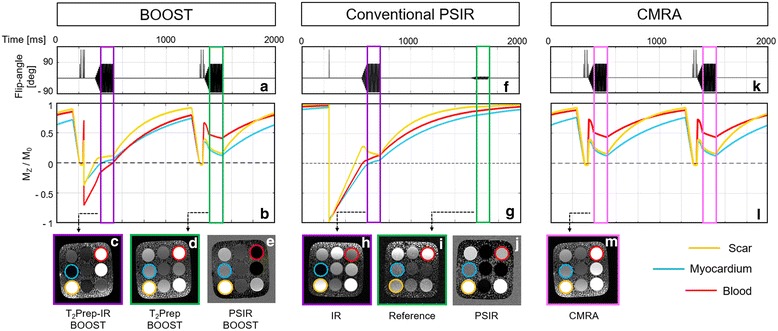
Sequence simulations and phantom images comparing BOOST and conventional sequences for LGE assessment and CMRA. Simulated magnetization of the post-contrast BOOST sequence (**a**, **b**), of a conventional PSIR sequence (**f**, **g**), and of a dedicated post-contrast CMRA sequence (**k**, **l**) are displayed. The expected longitudinal magnetization (M_z_/M_0_) is reported for blood (*red lines*), healthy viable myocardium (*blue lines*), and scar tissue (*orange lines*). Furthermore, results from the phantom experiments are displayed (BOOST: **c**, **d**, **e**; PSIR: **h**, **i**, **j**; CMRA: **m**) and the vial of interest are highlighted (blood – red circle, healthy viable myocardium – blue circle, and scar tissue – orange circle). Comparable contrast between the scar tissue and healthy viable myocardium can be observed in the PSIR reconstructions obtained with the BOOST sequence (PSIR BOOST) and the conventional PSIR sequence. Differently, improved contrast between the scar tissue and the healthy viable myocardium can be observed in the PSIR BOOST dataset when compared to the conventional PSIR sequence (phantom images in **e** and **j**). Data acquired with BOOST at even heartbeats (T_2_Prep BOOST, **d**) exhibit higher signal when compared to the reference image of the conventional PSIR sequence, acquired at a low flip-angle (**i**). In particular, T_2_Prep BOOST shows comparable signal and tissue contrast to that of a dedicated T_2_ prepared CMRA sequence (**m**)

Three different CMR sequences were simulated using the extended phase graphs (EPG) formalism [31]: 1) the BOOST sequence as illustrated in Fig. 1; 2) a conventional PSIR sequence for LGE assessment as described in [16]; 3) a dedicated T_2_-prepared bright-blood sequence for coronary CMRA [32]. The simulated tissue parameters were set as follows: healthy viable myocardium T_1_ = 550 ms, T_2_ = 45 ms; scar T_1_ = 300 ms, T_2_ = 45 ms; and post-contrast blood T_1_ = 450 ms, T_2_ = 200 ms. The T_1_ and T_2_ values of the tissues of interest were set to match those of the standardized phantom [33] used in this study, as described in the following paragraphs, and to approximate the properties of tissues about 15 min after gadolinium contrast injection [33, 34]. Accordingly, imaging parameters were set to match those of the performed phantom acquisitions. For both the BOOST and the conventional PSIR sequences, the TI was set to null the signal from healthy viable myocardium at odd heartbeats (corresponding to TI = 150 ms for the BOOST sequence and to TI = 350 ms for the conventional PSIR sequence). T_2_Prep duration was set to 40 ms for odd and even heartbeats of the BOOST sequence, and for the CMRA sequence. A flip-angle of 90 degrees was simulated for both the acquisition of the magnitude and the bright-blood reference image in the BOOST sequence as well as for the acquisition of the magnitude image in the conventional PSIR sequence. A flip-angle of 8 degrees was simulated for the reference image of the conventional PSIR sequence. For the CMRA sequence, a 90 degrees flip angle was simulated for each heartbeat. For all three sequences (BOOST, conventional PSIR, and CMRA), and for each individual heartbeat, a data acquisition duration equal to 120 ms was simulated, corresponding to 33 k-space lines. To minimize signal oscillations during acquisition, and to generate the iNAVs at each individual heartbeat, 14 bSSFP linear ramp-up pulses were simulated prior to imaging data acquisition. The heart-rate was simulated at 60 beats per minute with a total of 50 heartbeats (of which 2 were dummy heartbeats).

For all the simulated sequences, expected magnetization M_z_/M_0_ as well as absolute signal differences between the tissues of interest were computed. All the absolute signal differences (that are preserved in the final PSIR reconstruction) were computed at the beginning of the data acquisition using centric k-space ordering.

### Phantom experiments

#### Data acquisition

A standardized T_1_ and T_2_ phantom, with different vials resembling T_1_ and T_2_ values of the most relevant cardiac compartments [33], was used for data acquisition. Healthy myocardium, scar, and post-contrast blood T_1_ and T_2_ values were identical to the simulated ones. Data acquisition was performed using the BOOST sequence, the conventional PSIR sequence, and the dedicated CMRA acquisition. T_2_Prep durations, TIs, heart rate, number of k-space lines acquired per heartbeat and flip angle values were kept identical to those used in the simulations. Additional imaging parameters included: transverse orientation, Field of view (FOV) = 320x320x60 mm^3^, in-plane spatial resolution = 1 mm^2^, slice thickness = 2 mm, echo-time (TE) / repetition time (TR) = 1.56/3.6 ms, pixel bandwidth 977 Hz/pixel. The PSIR BOOST reconstruction was performed with and without intensity normalization for comparison purposes.

#### Data analysis

Signal to noise ratio (SNR) and CNR were quantified for the three sequences (BOOST, conventional PSIR, and dedicated CMRA). SNR of blood (SNR_blood_), healthy viable myocardium (SNR_myo_) and scar (SNR_scar_) were calculated for the odd heartbeats of BOOST and conventional PSIR, together with CNR between blood and healthy myocardium (CNR_blood-myo_), scar and blood (CNR_scar-blood_), and scar and healthy viable myocardium (CNR_scar-myo_). SNR_blood_ and CNR_blood-myo_ were quantified for even heartbeats for both the BOOST and the conventional PSIR sequence as well as for the dedicated CMRA acquisition. For the PSIR images obtained from the BOOST sequence (with and without intensity normalization) and the conventional PSIR sequence, CNR_blood-myo_, CNR_scar-blood_, and CNR_scar-myo_ were quantified after the removal of low spatial frequency signal components as described in [16, 35].

### In-vivo experiments

#### Data acquisition

Eighteen patients (52.7 ± 13.2 years, 9 males) who were referred for a clinical CMR examination were recruited for this study. In 16 out of 18 (89%) patients, a conventional 2D multi-slice and multi breath-hold bSSFP Cartesian PSIR sequence [16] was acquired in different orientations (four chamber view, three chamber view, short-axis view) starting 10 min after gadobutrol (0.2 mmol/kg) administration (Gadovist, Bayer, Berlin, Germany). Relevant imaging parameters for this acquisition include: FOV = 292 × 152 mm^2^, slice thickness = 8 mm, in plane spatial resolution = 1.4 mm^2^, 10 slices acquired for the short axis view, TE/TR = 1.26/2.9 ms, pixel bandwidth = 775 Hz/pixel, flip angle = 45 degrees, ECG triggering to the most quiescent diastolic period. The TI (typically ranging from 200 ms – 300 ms) was selected with a dedicated TI scout scan and was set to null the signal from the healthy viable myocardium. In 7 out of 18 (39%) subjects, a conventional free-breathing navigator-gated bright-blood whole-heart T_2_-prepared bSSFP (CMRA) Cartesian sequence was acquired after the breath-hold 2D PSIR sequences. For this acquisition, imaging parameters were set as follows: sagittal orientation, subject-specific FOV = 410x307x160–192 mm^3^, in plane spatial resolution = 1.4 mm^2^, slice thickness = 1.4 mm, TE/TR = 1.56/3.6 ms, pixel bandwidth = 575 Hz/pixel, flip angle = 90 degrees, T_2_Prep duration = 40 ms, 2× GRAPPA parallel imaging acceleration [36] with 24 calibration lines. Respiratory motion was compensated using diaphragmatic navigator gating and tracking (tracking factor equal to 0.6 [37]), with an acceptance window placed in end-expiration and with an amplitude equal to ±3.5 mm.

At the end of the clinical examination, the proposed ECG-triggered BOOST sequence was acquired under free-breathing and using the following imaging parameters: coronal orientation, in-plane spatial resolution = 1 mm^2^, slice thickness = 4 mm (interpolated to 2 mm during image reconstruction), subject-specific FOV = 320x320x80–130 mm^3^, TE/TR = 1.56/3.6 ms, pixel bandwidth 977 Hz/pixel. For both the magnitude image T_2_Prep-IR BOOST and the reference image T_2_Prep BOOST, the flip angle was set to 90 degrees and the T_2_Prep duration was equal to 40 ms. The subject-specific TI (typically ranging in the interval 100–180 ms) was selected to null the signal from viable healthy myocardium by acquiring a dedicated 2D BOOST TI scout scan during a breath-hold; such scout scan consisted of a magnetization-prepared cine sequence where a T_2_Prep-IR module is applied right after the R-wave at odd heartbeats, whereas T_2_-preparation solely is performed at the beginning of the cardiac cycle in even heartbeats. Cine frames belonging to the odd heartbeats were visually inspected to determine the optimized TI. In odd heartbeats, fat signal was suppressed for the effect of the inversion pulse (STIR-like approach) [38], while spectral pre-saturation (SPIR) [39] was used to suppress signal from epicardial fat at even heartbeats (Fig. 1). Images obtained in patients for whom BOOST data acquisition did not start later than 40 min after contrast injection were considered for further quantitative and qualitative data analyses. This temporal restriction was set to avoid a too pronounced washout of the contrast agent.

#### Quantitative data analysis

Quantitative data analysis was performed for the images acquired with the clinical 2D PSIR sequence, and for the 3D black-blood LGE and bright-blood CMRA datasets obtained with BOOST (PSIR BOOST and T_2_Prep BOOST, respectively). ROIs were manually drawn in blood, healthy viable myocardium and scar tissue (when present) at matching anatomical locations for both the clinical 2D PSIR and the 3D whole-heart PSIR BOOST images. Background noise was computed from a ROI with uniform signal positioned at the level of the liver, following the removal of low spatial frequency signal components as performed for the phantom images [16, 35]. CNR_blood-myo_, CNR_scar-blood_ and CNR_scar-myo_ were quantified for both the clinical 2D PSIR and the 3D whole-heart PSIR BOOST images. CNR values were quantified in the subjects for whom both the 2D PSIR and BOOST sequences were acquired, and compared using a paired 2-tailed Student t-test. *P* = 0.05 was set as the threshold to determine statistical significance. Acquisition times of all the acquired sequences (2D PSIR acquisition – including pauses between breath-holds –, clinical whole-heart CMRA, and BOOST) were recorded. In addition, scan efficiency was recorded for the clinical CMRA acquisition with diaphragmatic navigator gating.

#### Qualitative data analysis

All the datasets (3D black-blood PSIR BOOST, 3D bright-blood T_2_Prep BOOST, conventional 3D CMRA acquisition, and clinical 2D PSIR) were anonymized and stored in a randomized order. Qualitative grading of the anonymized images was performed by two experienced cardiologists (T.F.I. and I.R., *SCMR III* certification) blinded to clinical data. For all the LGE images (black-blood 3D PSIR BOOST and 2D clinical PSIR), presence and location of LGE were assessed. Furthermore, images were graded in terms of diagnostic quality on consensus basis using a 4-point scale system where *1* indicates a fully diagnostic dataset without the presence of artefacts, *2* indicates a diagnostic dataset with only minor artefacts present, *3* indicates a diagnostic dataset with significant artefacts, and *4* indicates an artefacts-rendering images non-diagnostic dataset. Subjective scores for LGE visualization were compared with a paired Wilcoxon signed-rank test to assess statistical differences; *P* < 0.05 was considered statistically significant. Statistical analyses were performed considering the cases for whom both the clinical 2D PSIR and the BOOST sequences were acquired. For all acquired bright-blood datasets (conventional 3D CMRA and T_2_Prep BOOST), the ability to identify the origin and the proximal course of the coronary arteries was graded for four relevant coronary segments: left main (LM), left anterior descending coronary artery (LAD), left circumflex coronary artery (LCX), and right coronary artery (RCA). Grading was performed on consensus basis using a 4-point scale system as that used for the grading of LGE datasets.

## Results

All data acquisitions and reconstructions were carried out successfully and all quantified endpoints are reported hereafter.

### Sequence simulations

All simulated pulse sequences (proposed post-contrast BOOST sequence, conventional PSIR sequence [16], and dedicated post-contrast CMRA acquisition [32]) and the resulting steady state magnetization behavior for blood, myocardium and scar tissues are reported in Fig. 2. For the T_2_Prep-IR BOOST sequence (odd heartbeats) the expected magnetization M_z_/M_0_ of the healthy viable myocardium, blood and scar varied within the intervals 0.000–0.059, −0.158 – 0.003, and 0.108–0.116, respectively, during imaging data acquisition. This resulted in an absolute signal difference between myocardium and scar of +0.108 and of +0.266 between blood and scar. For the conventional PSIR sequence, and in correspondence to the acquisition of the magnitude image, the expected magnetization M_z_/M_0_ of the healthy viable myocardium varied within the interval − 0.002 – 0.058, whereas the expected magnetization M_z_/M_0_ of blood and scar varied within the intervals 0.056–0.146 and 0.207–0.144, respectively. This resulted in an absolute signal difference between healthy myocardium and scar amounting to +0.208, and of +0.151 between blood and scar. For the conventional PSIR sequence, the reference image at even heartbeats (low flip-angle) exhibited high values of expected magnetization M_z_/M_0_, varying in the intervals 0.807–0.858, 0.877–0.919 and 0.951–0.967, for healthy viable myocardium, blood, and scar tissue, respectively. Conversely, even heartbeats of the BOOST sequence (T_2_Prep BOOST) exhibited reduced expected magnetization M_z_/M_0_ for the tissues of interest, since data acquisition is performed with higher flip-angle and is preceded by a T_2_Prep. Specifically, the expected magnetization M_z_/M_0_ of healthy viable myocardium, blood and scar varied within the intervals 0.230–0.123, 0.490–0.420 and 0.267–0.167, respectively. This resulted in a blood/myocardium ratio of 2.13, which is adequate for anatomy and coronary lumen visualization. Similarly, for the conventional post-contrast CMRA sequence, the expected magnetization M_z_/M_0_ of healthy viable myocardium, blood and scar varied within the intervals 0.220–0.100, 0.498–0.438, and 0.267–0.161, respectively, leading to a blood/myocardium ratio equal to 2.26.

### Phantom experiments

**Fig. 3:**
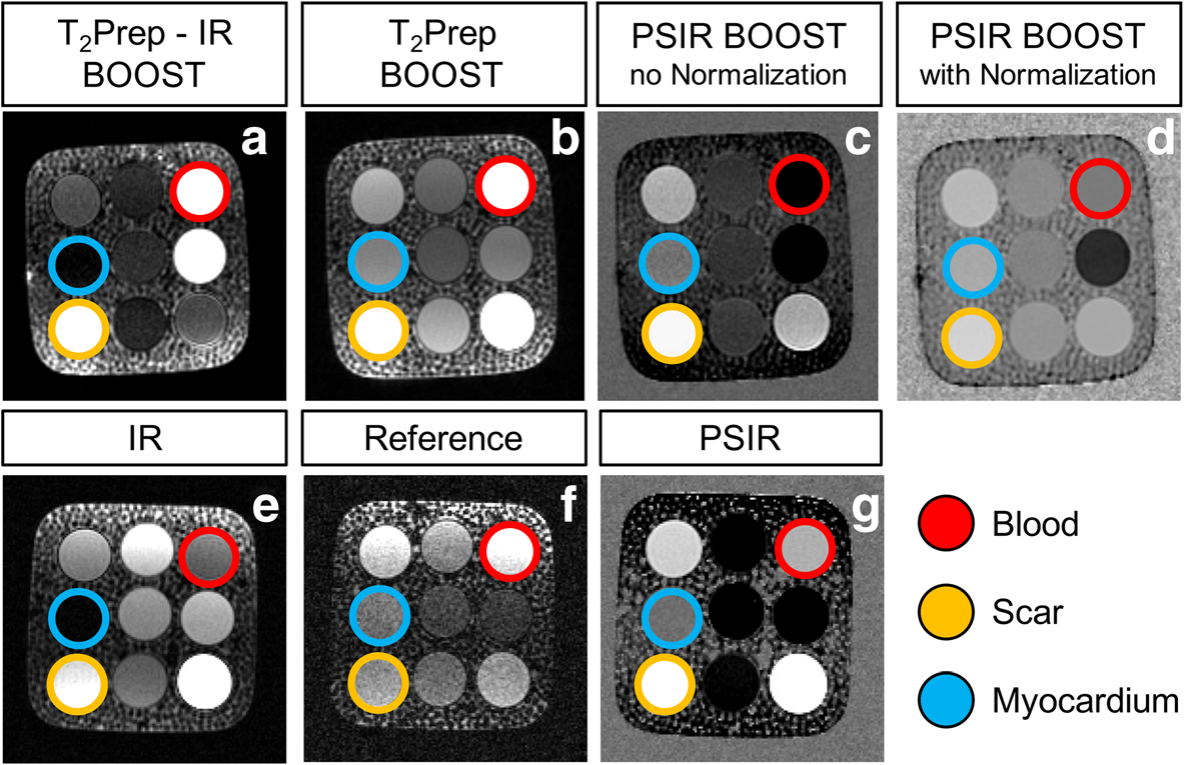
Phantom images obtained with the BOOST and the conventional PSIR sequence. Imaging data were acquired by nulling the signal from the healthy viable myocardium (*blue vial*) in the magnitude images (**a**, **e**). Differently from **f**, the T_2_Prep BOOST dataset, acquired at a high flip-angle, exhibits both high signal from the blood (*red vial*) and pronounced contrast between blood and healthy viable myocardium (**b**). The PSIR reconstruction obtained with BOOST and using intensity normalization (**d**) shows reduced tissue contrast, which is restored once intensity normalization is not applied (**c**). Furthermore, such restored contrast between the scar tissue (*orange vial*) and the healthy viable myocardium is comparable to that of the PSIR reconstruction in (**g**), while improved contrast between scar and blood can be appreciated

Phantom images obtained with the proposed post-contrast BOOST sequence, the conventional PSIR sequence [16], and the dedicated post-contrast CMRA acquisition are shown in Fig. 2. ROIs corresponding to post-contrast blood, healthy viable myocardium, and scar are indicated by red, blue and yellow circles, respectively. All the endpoints that were quantified for the phantom acquisitions are summarized in the Additional file 1. The T_2_Prep-IR BOOST phantom dataset showed strong signal from both blood and scar tissue, while providing suppression of the signal belonging to the vial mimicking the viable myocardium. The magnitude image of the conventional PSIR acquisition showed effective suppression of signal from the viable myocardium, and high signal from the vial mimicking the scar. The reference image T_2_Prep BOOST, designed for the visualization of the heart anatomy, great vessels, and coronary lumen, showed SNR_blood_ and CNR_blood-myo_ comparable to those provided by the dedicated CMRA acquisition. Conversely, the reference image of the conventional PSIR acquisition (acquired at a low flip angle) showed reduced SNR_blood_ and CNR_blood-myo_. The PSIR reconstructions obtained using the BOOST sequence with and without normalization are displayed in Fig. 3. Reduced tissue contrast is observed for the PSIR reconstruction with intensity normalization. Conversely, tissue contrast was restored in the PSIR reconstruction without intensity normalization. The PSIR BOOST phantom dataset (obtained without intensity normalization) showed effective blood signal suppression, leading to improved CNR_scar-blood_ when compared to the more conventional PSIR sequence.

### In-vivo experiments

**Fig. 4:**
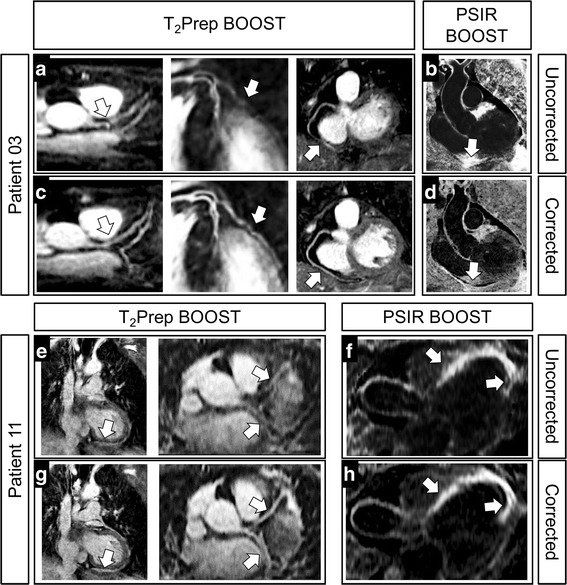
Improvement in BOOST image quality after translational motion correction in two representative patients. The use of translational motion correction along the SI and RL directions reduces blurring artefacts and improves coronary vessel sharpness in the bright-blood T_2_Prep BOOST datasets (arrows in **a**, **c**, **e**, and **g**). Motion compensation recovers also small details as showed in the zoomed images. Furthermore, improved image sharpness can be observed on the black-blood PSIR-like reconstructions (arrows in **b**, **d**, **f**, and **h**), where a sharper delineation of the LGE uptake can be appreciated following motion correction (**f** versus **h**)

Quantitative and qualitative data analysis was performed for 12 of 18 patients for whom BOOST data acquisition started less than 40 min after contrast agent injection. The average time after injection for the BOOST datasets was 27:47 ± 3:35 min. Among those patients, the acquisition of the clinical 2D PSIR sequence and of the clinical 3D whole-heart CMRA with diaphragmatic navigator was performed in 10 and 6 cases, respectively. Clinical, imaging, and demographic characteristics are summarized in Table 1. Acquisition times were 7:48 ± 4:03 min for the clinical 2D PSIR sequence (including pauses between breath-holds), 13:06 ± 3:05 min for the conventional CMRA acquisition with diaphragmatic navigator (with an average scan efficiency of ~ 47%), and 12:07 ± 1:56 min for data acquisition with BOOST that provides both black-blood LGE and bright-blood anatomical images. For the BOOST sequence, image-based navigation enabled data acquisition with 100% scan efficiency (i.e., none of the acquired data was discarded during image reconstruction) and predictable scan time. Furthermore, translational motion correction led to effective respiratory motion correction for both the bright-blood T_2_Prep and the black-blood PSIR BOOST datasets in most cases, as shown in Fig. 4 for two representative patients.

**Table 1:** Summary of patients’ data used for quantitative analysis

	Gender	Age (years)	Heartbeats per minute	Clinical Condition	LGE findings	3D whole heart CMRA
Patient 01	F	25	85	Atrial fibrillation	No	Yes
Patient 02	F	30	70	Atrial fibrillation (previous myocarditis)	No	No
Patient 03	F	48	70	Eosinophilic Granulomatous Polyangitis (Churg-Strauss syndrome)	No	No
Patient 04	M	50	80	Myocarditis	Yes Diffuse mid-wall	No
Patient 05	M	67	55	Myocardial infarction	Yes Transmural	No
Patient 06	M	56	65	Advanced Hypertensive heart disease	No (no 2D PSIR)	Yes
Patient 07	M	66	70	Myocardial infarction	Yes Transmural and subendocardial	No
Patient 08	M	62	80	Myocardial infarction	Yes Mid-wall	Yes
Patient 09	M	59	45	Myocardial infarction	Yes Subendocardial	Yes
Patient 10	F	30	75	Myocardial infarction	Yes Subendocardial	Yes
Patient 11	F	74	80	Myocardial infarction	Yes Transmural	No
Patient 12	M	52	75	Suspected myocardial infarction	No (no 2D PSIR)	Yes

Furthermore, the presence of LGE findings is stated (“no 2D PSIR” pertains the cases where 2D PSIR acquisition was not performed and presence of LGE uptake was assessed with BOOST only). In addition, it is indicated whether the acquisition of the conventional, 3D whole-heart CMRA sequence was performed or not

#### Quantitative data analysis

**Fig. 5:**
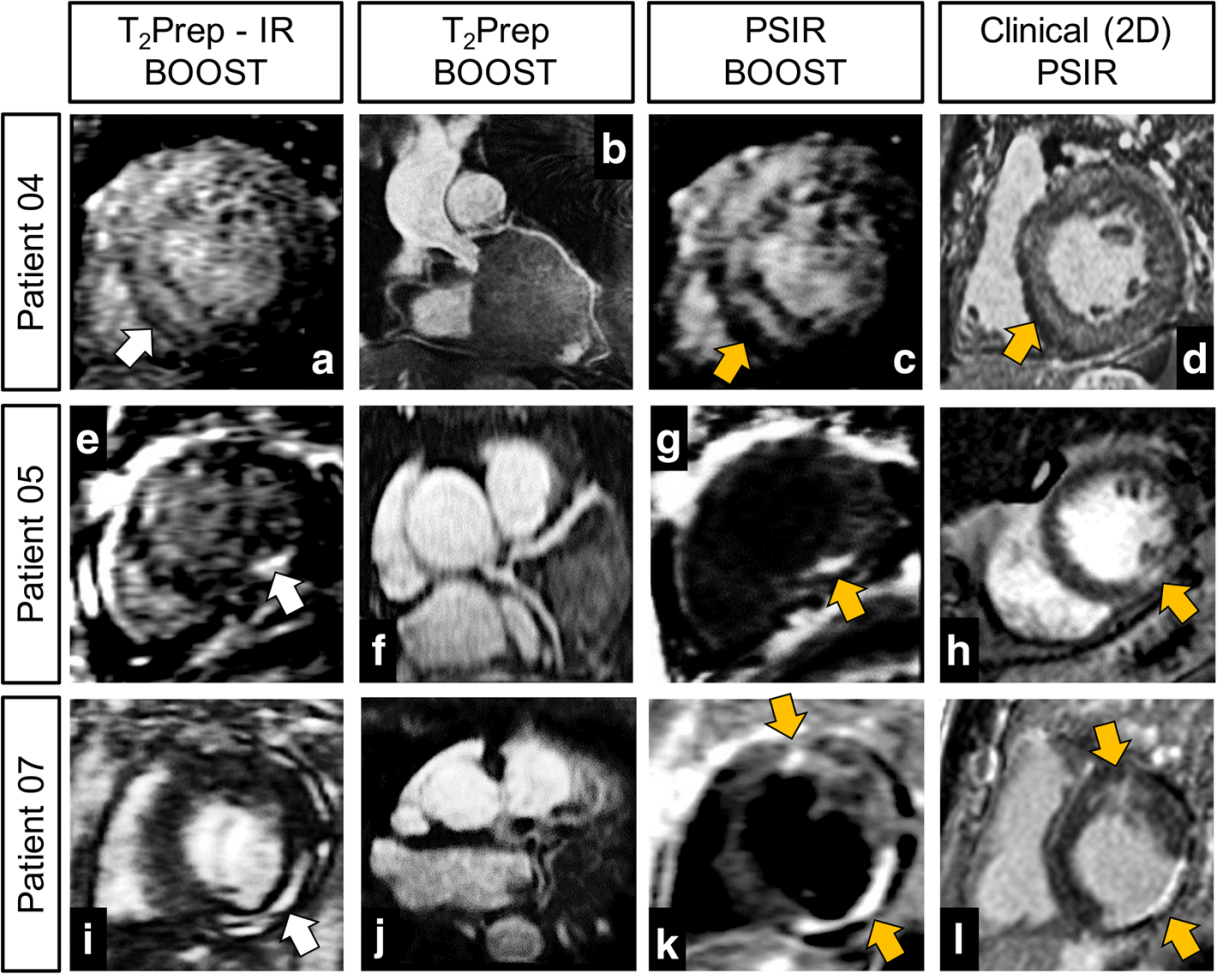
Comparison between the proposed 3D whole-heart BOOST framework and the clinical 2D PSIR acquisition. Images in **a**, **e**, and **i** show the LGE uptake as depicted in the T_2_Prep-IR BOOST datasets (*white arrows*), where signal from the blood pool is present and the viable myocardium is suppressed. Reformats in **b**, **f**, and **j** show the coronary reformats obtained from the 3D whole-heart bright-blood T_2_Prep BOOST dataset. Complementary 3D black-blood LGE images obtained with BOOST are shown in **c**, **g**, and **k**. All the images from the T_2_Prep-IR BOOST and the PSIR BOOST datasets were reformatted to match the orientation of the clinical 2D PSIR acquisitions (**d**, **h**, and **l**). The LGE uptake identified in both the T_2_Prep-IR BOOST and PSIR BOOST datasets matches that of the clinical 2D PSIR acquisition. Furthermore, improved contrast between the scar tissue and the blood pool can be appreciated in the 3D PSIR BOOST datasets when compared to the 2D PSIR acquisition (**g**, **k** versus **h**, **l**, *orange arrows*). LGE uptake appears more shallow and blood pool signal is not entirely suppressed in Patient 04 with myocarditis (**c**, **d**, *orange arrows*), due to a longer TI

The endpoints quantified for the conventional 2D PSIR sequence amounted to CNR_blood-myo_ = 15.2 ± 8.1, CNR_blood-scar_ = 4.1 ± 5.6 and CNR_scar-myo_ = 12.3 ± 9.3. The black-blood PSIR BOOST datasets presented effective nulling of the blood signal, leading to significantly improved CNR_blood-scar_ (equal to 15.8 ± 3.3, *P* < 0.025) and significantly reduced CNR_blood-myo_ (4.2 ± 3.6, *P* < 0.001) when compared to the clinical 2D PSIR sequence (Fig. 5). Quantified CNR_scar-myo_ was equal to 13.02 ± 4.56 (P = NS in comparison to the clinical 2D PSIR sequence).

#### Qualitative data analysis

**Fig. 6:**
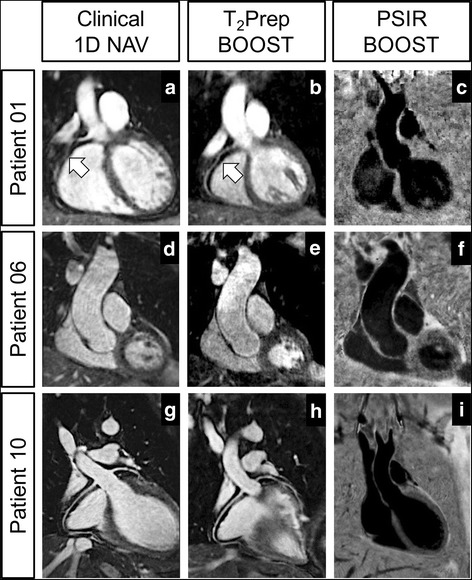
Comparison between conventional 3D whole-heart acquisition with diaphragmatic navigator and the proposed bright-blood T_2_Prep BOOST. Improved delineation of the RCA can be appreciated in Patient 01 with T_2_Prep BOOST when compared to the conventional CMRA acquisition (arrows in **a**, **b**). Dilated aorta can be observed in Patient 06 due to the presence of hypertensive heart disease (**d**, **e**). Excellent coronary delineation was obtained with both sequences in Patient 10 (**g**, **h**). Furthermore, the complementary black-blood PSIR BOOST datasets for LGE assessment are shown in **c**, **f**, and **i**

Eleven of 12 3D PSIR BOOST datasets were considered diagnostic, with an average grade of 1.75 ± 1.21; specifically, 8/12 cases were graded *1*, one single case was graded *2*, two cases – where incomplete blood suppression was observed – were graded *3*, and one single case was graded *4* due to the presence of residual motion and pronounced artefacts originating from the rigid translation of the chest wall and arms. Complete correspondence between clinical 2D PSIR acquisitions and 3D PSIR BOOST datasets was found in 8/10 cases in terms of LGE findings and location of the LGE uptake. In two individual cases (Patient 08 and Patient 10) LGE was not visible in the 3D PSIR BOOST dataset. Both datasets were graded *1*, meaning no residual artefacts were visible and optimal blood signal suppression was achieved. In these two cases, however, BOOST data acquisition started 39:57 min and 39:32 min after contrast injection, following a conventional 3D CMRA acquisition (duration 17:04 min and 11:31 min) that was performed between the 2D clinical PSIR and the 3D BOOST sequences. This particularly pronounced delay between the two LGE acquisitions, together with the absence of residual artefacts and the achievement of adequate blood signal suppression in the PSIR BOOST datasets, suggests that contrast agent washout prevented adequate scar depiction. Clinical 2D PSIR images were graded 1 for all the 10 patients where the sequence was acquired. No statistically significant difference was found in terms of visual grading (P = NS) when comparing the 3D PSIR BOOST datasets and the clinical 2D PSIR counterpart (average grade of BOOST in these 10 patients: 1.80 ± 1.31). In terms of coronary conspicuity, image quality scores evaluated by consensus grading were 1.50 ± 1.22 (LM), 2.00 ± 1.54 (LAD), 2.50 ± 1.64 (LCX) and 2.83 ± 1.16 (RCA) for the conventional CMRA with diaphragmatic navigator (6 patients, Table 1). A trend of improvement in terms of coronary delineation was quantified with BOOST in the same 6 patients; for those subjects, LM and LAD were graded *1* in all cases. For LCX and RCA, average grades were equal to 1.50 ± 1.22 and 1.50 ± 0.83, respectively. A visual comparison between T_2_Prep BOOST datasets and conventional CMRA is shown in Fig. 6. Overall visual grading obtained with T_2_Prep BOOST (considering the entire cohort of 12 patients, which includes cases where the acquisition of the conventional CMRA was not performed) was 1.07 ± 0.27 for the LM, 1.07 ± 0.27 for the LAD, 1.07 ± 0.27 for the LCX, and 1.53 ± 0.77 for the RCA.

**Fig. 7:**
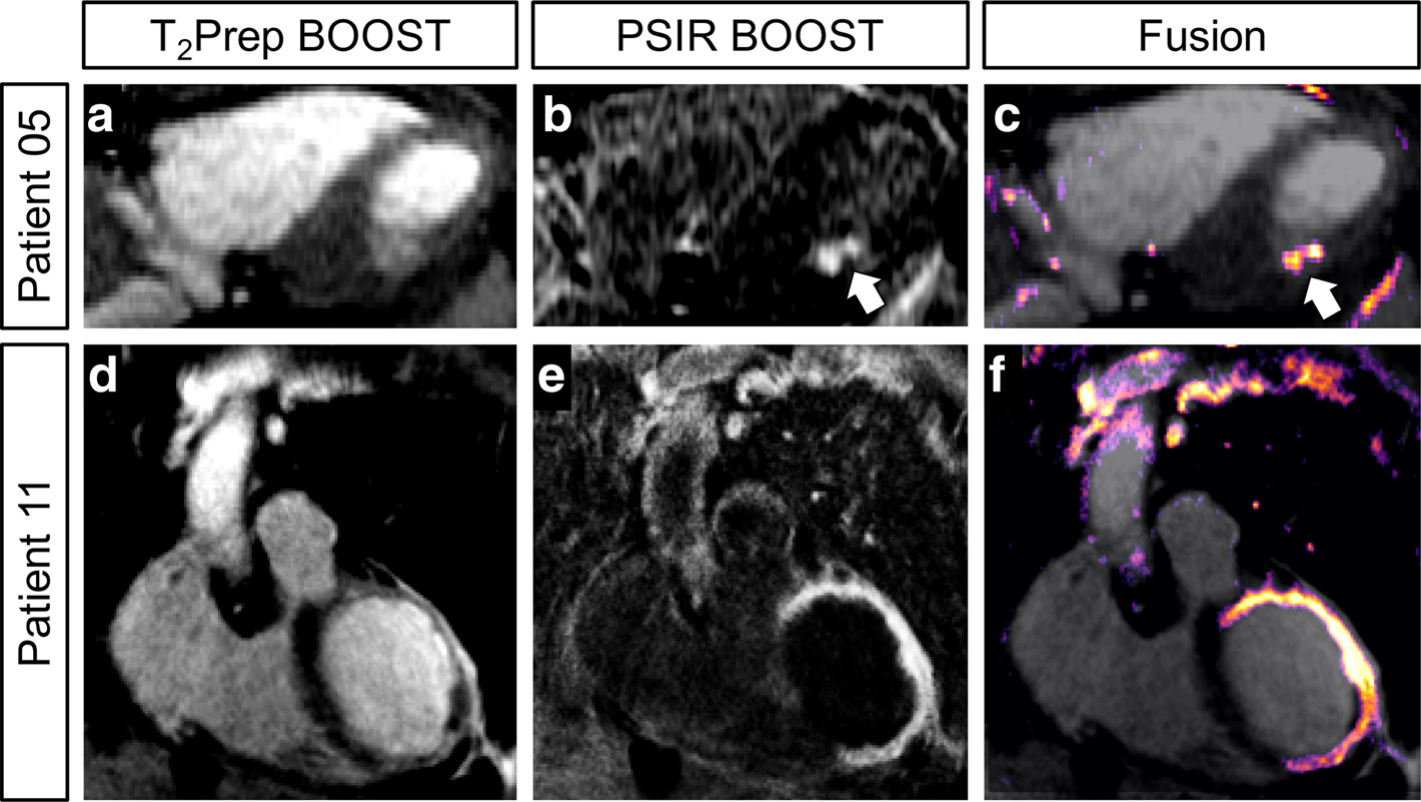
Fusion of the bright-blood T_2_Prep BOOST and the black blood PSIR BOOST datasets. Images correspond to two representative patients with positive LGE findings. Bright-blood images for visualization of the heart anatomy are shown in **a**, **d** **(T**_**2**_ **Prep-BOOST)**. Complementary visualization of scar tissue (PSIR BOOST) is shown in **b**, **e**; these datasets could potentially be used for an easy scar segmentation, as unclear border between the surrounding tissues and the scar itself have disappeared. Fusion images, where the anatomical localization of the scar can be retrieved, are shown in **c**, **f**

Fusion of a bright-blood T_2_Prep BOOST with a black-blood PSIR BOOST datasets (obtained with the Horos software, V1.1.7) is illustrated in Fig. 7 to demonstrate the location and transmurality of the infarct obtained from the black-blood PSIR dataset.

## Discussion

In this study, we extended the use of a novel PSIR-like framework, referred to as BOOST, to post-contrast applications for simultaneous 1) black-blood LGE assessment and 2) bright-blood heart anatomy, great vessels, and coronary lumen visualization. With the BOOST framework, the acquisition of the magnitude image (T_2_Prep-IR BOOST) is based on a T_2_Prep-IR module for optimal contrast between the blood pool and scar tissue after PSIR computation (black-blood PSIR BOOST). Furthermore, the acquisition of the reference image (bright-blood T_2_Prep BOOST) is performed with a high flip-angle and it is preceded by a T_2_Prep module. This ensures adequate signal and tissue contrast for the visualization of heart anatomy, great vessels, and the coronary lumen. In contrast to previously published approaches providing a single bright-blood dataset for the simultaneous visualization of LGE and proximal coronary arteries [40], our framework generates two separate yet co-registered 3D volumes, each one being specifically designed and optimized for the visualization of the coronary lumen (bright-blood T_2_Prep BOOST) and myocardial scar (black-blood PSIR BOOST).

Sequence simulations and phantom acquisitions showed that the proposed post-contrast PSIR BOOST dataset achieves improved scar-blood contrast when compared to a more conventional PSIR sequence for LGE imaging [16]; this was confirmed by in vivo measurements in patients. While the PSIR BOOST volume provided adequate LGE depiction in most of the patients with positive findings, phantom experiments indicate higher CNR_scar-myo_ in the T_2_Prep-IR BOOST datasets, where precise viable myocardial nulling is achieved; this can be qualitatively appreciated in vivo as shown in Fig. 5. As such, referring to the T_2_Prep-IR BOOST dataset for the detection of subtle, non-ischemic, fibrosis patterns might be preferable; this aspect, however, needs further investigation and will be analyzed in future studies. Furthermore, sequence simulations show that the bright-blood T_2_Prep BOOST dataset provides SNR_blood_ and CNR_blood-myo_ similar to those of a dedicated T_2_-prepared post-contrast CMRA acquisition. In vivo acquisitions showed that respiratory motion corrected bright-blood T_2_Prep BOOST datasets allowed visualization of the origin and the proximal course of the coronary arteries (LM, LAD, LCX, and RCA) with high diagnostic quality. A trend of improvement was observed in comparison to the conventional CMRA; respiratory motion compensation performed with diaphragmatic navigator gating assumes a fixed linear correlation between the respiratory motion of the liver and that of the heart. The fixed correlation factor of 0.6 [37] that was used in this study might have been inexact for some of the subjects, thus leading to sub-optimal motion compensation. Conversely, with the use of image-based navigation, respiratory motion information can be directly extracted from the heart itself, thus avoiding the risk of imprecise approximations. In addition, with image-based navigation, it is possible to correct for movements along both SI and RL directions [27]. These aspects may have been a contributing factor of the improved coronary delineation that was obtained with BOOST. Furthermore, and as predicted by sequence simulations, the black-blood PSIR BOOST reconstruction provided visualization of LGE with diagnostic quality in most cases and significantly improved CNR_scar-blood_ was quantified in comparison to clinical 2D PSIR acquisitions [16].

The PSIR reconstruction performed with the proposed framework exactly follows that described in [16], with the exception of the intensity normalization step that is conventionally performed at the end of the PSIR pipeline. In contrast to previously published post-contrast PSIR sequences [16], the reference image (T_2_Prep BOOST) acquired in our approach exhibits high tissue contrast, thus preventing the application of surface coil intensity normalization. In fact, the presence of high tissue contrast in the reference image significantly alters the resulting contrast of the normalized PSIR reconstruction (Fig. 3). The use of surface coil intensity normalization is typically exploited to compensate for large variations in the intensity of the image caused by rapid fall-off of the surface-coil fields, thus improving the local tissue contrast. This was shown to be particularly beneficial for the visualization of subendocardial infarcts, given the fact that the contrast between scar tissue and blood is particularly reduced in conventional PSIR acquisitions [16]. With this new sequence configuration, however, intrinsically enhanced contrast between blood and scar tissue is provided using a T_2_Prep-IR module for the acquisition of the magnitude image; in addition, the use of pre-scan based normalization readily available on commercial scanners can be exploited to compensate for variations in signal intensity. This might alleviate the need for surface coil intensity normalization, however further validation may be needed to corroborate this point.

The integration of the framework with image-based navigation enabled data acquisition during free-breathing with 100% scan efficiency and predictable scan time. The acquisition time for BOOST (approximately 12 min) was similar to that of a conventional CMRA acquisition with diaphragmatic navigator (approximately 13 min), considering an average scan efficiency of 50% and 2× parallel imaging acceleration. The BOOST framework, however, provides both a bright- and black-blood dataset in the same acquisition time, whereas the overall acquisition of conventional CMRA and 2D PSIR sequences was about 20 min in our cohort of patients. This intrinsic efficiency of the BOOST framework holds potentials for reducing the scan time that is currently needed to perform a complete CMR examination. This might be particularly beneficial in the case of claustrophobic, anxious, or clinically unstable patients. Additionally, reducing the overall examination time would imply economic benefits and reduction of patients waiting lists. Future technical developments of the BOOST sequence will include the integration of acceleration techniques [36, 41, 42] to improve both the nominal acquisition time as well as the spatial resolution. Furthermore, improvements in the acquired spatial resolution might enable isotropic acquisitions that would, for instance, allow for more robust visualization of the mid and distal coronary arteries. Similarly, the achievement of higher spatial resolution could benefit tissue characterization, allowing for a more accurate delineation of scar tissue and enabling a more accurate image fusion between the bright-blood T_2_Prep BOOST and the black-blood PSIR BOOST datasets for the assessment of scar location and transmurality. Additionally, the framework will be integrated with algorithms for arrhythmia rejection that could further improve the image quality that was obtained in this study. Currently, BOOST is combined with image-based navigation enabling in-line translational motion correction along the SI and RL directions. However, the breathing pattern in patients is often more complex and involves translation, rotation, and non-rigid deformations [43–45]. Therefore, future technical developments will aim at combining the BOOST framework with strategies for non-rigid respiratory motion correction [46] that might be particularly beneficial in very sick patients who often have irregular breathing patterns [47]. In addition, the use of non-rigid respiratory motion correction may help to reduce ghosting artefacts that may originate from rigid translation of static tissues such as the chest wall and arms during the motion correction process. Similarly, a rigid registration between the T_2_Prep-IR BOOST dataset and the T_2_Prep BOOST dataset is currently performed prior PSIR computation to compensate for residual mis-registration errors; this may be also sub-optimal and the use of non-rigid registration could further improve the quality of the resulting PSIR BOOST dataset and additionally reduce the risk of phase errors that may originate in portions of the image where phases are not varying smoothly (e.g. in correspondence to the interface between different tissues).

In this study, the BOOST acquisition was performed at the end of a clinical CMR examination as it was considered unethical to potentially jeopardize the acquisition of conventional LGE data at the expense of a novel sequence at this stage. Injection timing was optimized to provide optimal contrast agent concentration during the acquisition of the clinical 2D PSIR sequences, thus providing suboptimal contrast conditions for the BOOST scan. This was noticed particularly in two specific cases (Patient 08 and Patient 10), where LGE uptake could not be depicted despite the absence of motion artefacts and the achievement of optimal blood signal suppression. Furthermore, as the BOOST acquisition was performed at the end of the clinical scan, there may have been more respiratory or heart-rate irregularities that might have had an additional detrimental effect on the image quality that was obtained with BOOST. However, scan time was not prolonged by more than 15 min at most. Therefore, future studies are warranted to rigorously compare the proposed post-contrast 3D BOOST sequence and conventional 2D PSIR acquisitions by, for instance, randomizing the order of the two acquisitions and by performing separate Gd injections to ensure equivalent contrast conditions. Kellman et al. [19] demonstrated that black-blood LGE provides improved conspicuity of subendocardial infarcts; future studies will aim at investigating the accuracy of black-blood PSIR BOOST for the quantification of scar transmurality and, thus, regional viability assessment. Furthermore, accuracy in the detection and quantification of ischaemic scar will be validated. Similarly, further clinical validation of BOOST is needed in comparison to conventional CMRA in patients with angiographically confirmed coronary artery disease.

The improved contrast between blood pool and scar provided by the proposed black-blood PSIR BOOST images may facilitate scar segmentation. However, the nulling of the blood and viable myocardium signal reduces the depiction of the heart anatomy and might challenge the localization of the scar itself. This challenge can be addressed by fusing the co-registered black blood PSIR dataset with the bright blood whole heart dataset, which then allows both scar and myocardial anatomy visualization as shown in Fig. 7. This characteristic makes this framework particularly suitable for the planning of electrophysiology procedures. Similarly, the framework may be beneficial for the visualization of lesions after ablation and follow-up of patients. This further enlarges the spectrum of potential clinical applications of post-contrast BOOST, that will be tested in dedicated studies in the upcoming future.

## Conclusions

We demonstrated the feasibility of simultaneous black-blood LGE imaging and bright-blood visualization of cardiac anatomy, the great vessels, and the coronary artery lumen using a novel motion corrected multi-contrast 3D imaging sequence, referred to as BOOST. Data acquisition with BOOST was performed in free-breathing and ensures whole-heart coverage, 100% scan efficiency, and predictable scan time. The framework was validated in a group of cardiac patients and showed high quality depiction of the coronary arteries in comparison to standard CMRA and good agreement with 2D PSIR LGE scar visualization. This novel sequence has a broad spectrum of potential clinical applications.

## Additional file

Additional file 1: Table summarizing the quantified endpoints for all the performed phantom acquisitions. (DOCX 14 kb)

## Abbreviations

BOOST: Bright-blood and black-blood phase sensitive inversion recovery
bSSFP: Balanced steady state free precession
CMR: Cardiovascular magnetic resonance
CMRA: Coronary magnetic resonance angiography
CNR: Contrast to noise
EPG: Extended phase graph (simulation)
FOV: Field of view
Gd: Gadolinium
GRAPPA: Generalized autocalibrating partially parallel acquisition
iNAV: Image-based navigator
IR: Inversion recovery
LAD: Left anterior descending coronary artery
LCX: Left circumflex coronary artery.
LGE: Late gadolinium enhancement
LM: Left main coronary artery
PSIR: Phase-sensitive inversion recovery
RCA: Right coronary artery
ROI: Region of interest
SNR: Signal to noise
T_2_Prep: T_2_-prepared
T_2_Prep-IR: T_2_-prepared inversion recovery\
TE: Echo time
TI: Inversion time
TR: Repetition time
