# Supplementary material for: 3D whole-heart phase sensitive inversion recovery CMR for simultaneous black-blood late gadolinium enhancement and bright-blood coronary CMR angiography
Source: J Cardiovasc Magn Reson. 2017 Nov 27;19:94. doi: 10.1186/s12968-017-0405-z (PMC5702978; doi:10.1186/s12968-017-0405-z)
Supplement: Additional file 1: — Table summarizing the quantified endpoints for all the performed phantom acquisitions. (DOCX 14 kb) [file 12968_2017_405_MOESM1_ESM.docx]

|  | **BOOST** | | **Conventional PSIR** | **CMRA** |
| --- | --- | --- | --- | --- |
|  | **T_2_Prep – IR BOOST** | | **Magnitude Image** |  |
| SNR blood: | 79.2 | | 34.9 |  |
| SNR myocardium: | 5.4 | | 2.1 |  |
| SNR scar: | 87.6 | | 77.6 |  |
| CNR blood-myocardium: | 73.8 | | 32.8 |  |
| CNR scar-blood: | 8.4 | | 42.6 |  |
| CNR scar-myocardium: | 82.2 | | 75.5 |  |
|  | **T_2_Prep BOOST** | | **Reference Image** |  |
| SNR blood: | 204.2 | | 24.4 | 218.0 |
| CNR blood-myocardium: | 122.7 | | 5.9 | 130.0 |
|  | **PSIR BOOST** | | **PSIR** |  |
|  | With Normalization | Without Normalization | With Normalization |  |
| CNR blood-myocardium: | 2.7 | 63.2 | 18.4 |  |
| CNR blood-scar: | 5.1 | 120.7 | 43.8 |  |
| CNR scar-myocardium: | 2.4 | 57.5 | 62.1 |  |
